# Supplementary material for: Deep learning image analysis for filamentous fungi taxonomic classification: Dealing with small datasets with class imbalance and hierarchical grouping
Source: Biol Methods Protoc. 2024 Aug 27;9(1):bpae063. doi: 10.1093/biomethods/bpae063 (PMC11387011; doi:10.1093/biomethods/bpae063)
Supplement: bpae063_Supplementary_Data [file bpae063_supplementary_data.zip › Figure_Captions.docx]

**Figure 1.** Example of a scanned image of cultures in the dataset (upper). Circles denote Petri dish object detection using the Circle Hough Transform. A zoom in on the bottom-left sample as an example of a filamentous fungus (lower).

**Figure 2.** Absolute number of isolates assigned to a known order by DNA extraction and Sanger sequencing method. Fungal isolates were extracted from soil samples, and the cultures were bred in Petri dishes. Taxonomic identification was annotated using blast+ classification algorithm, querying isolate ITS region to UNITE database.

**Figure 3.** (a) Number of unique taxa/classes according to taxonomic rank for 606 samples. (b-g) Frequency distribution within each taxonomic rank. C is the number of categories, MC+ is the majority class count, MC- minority class count. A category is regarded as majority if the number of samples # is higher than the average number of samples per category. LriD is the likelihood ratio imbalance degree. For phylum, class, and order levels the majority classes are labeled for visualization.

**Figure 4.** Performance for Separate Local per-level classifiers (SL) finetuned in 20 epochs according to Matthews Correlation Coefficient on (a) original, (b) naive oversampled, (c) transform oversampled data and according to Accuracy on (d) original, (e) naive oversampled, (f) transform oversampled data sets.

**Figure 5.** Comparison of best test performances each model achieved according to (a) Matthews Correlation Coefficient and (b) Accuracy on 606 samples.

**Figure 6.** Confusion Matrix of Separate Local per-level classifiers (SL) trained on 606 samples of naïve oversampled dataset, showing observed vs predicted taxonomic group. Prediction on test data for taxonomic ranks (a) phylum at epoch 17, (b) class at epoch 14, and (c) order at epoch 11.

**Figure 7.** LIME explanation for *Apiotrichum dulcitum* predicted with ML at epoch 4, with neighborhood size 1000 and 100 superpixels. Segmentation is performed by quickshift algorithm with kernelsize 6, max distance 50 and ratio 0.5. Black highlighted areas are LIME explanations at rank (a) phylum, (b) class, (c) order, (d) family, (e) genus and (f) species.

**Supplementary**

**Figure S1.** Performance for Separate Local per-level classifiers (SL) finetuned in 20 epochs according to Matthews Correlation Coefficient on (a) original, (b) naive oversampled, (c) transform oversampled data and according to Accuracy on (d) original, (e) naive oversampled, (f) transform oversampled data sets.

**Figure S2.** Multi-Label classifier (ML) finetuned in 20 epochs according to Matthews Correlation Coefficient on (a) original, (b) naive oversampled, (c) transform oversampled data and according to Accuracy on (d) original, (e) naive oversampled, (f) transform oversampled data sets.

**Figure S3.** Hierarchically-Chained Local per-level classifiers (HC) finetuned in 20 epochs according to Matthews Correlation Coefficient on (a) original, (b) naive oversampled, (c) transform oversampled data and according to Accuracy on (d) original, (e) naive oversampled, (f) transform oversampled data sets.

**Figure S4.** Stability for Separate Local per-level classifiers (SL) on the 606 sample set, finetuned in 20 epochs according to Matthews Correlation Coefficient on (a) original, (b) naive oversampled, (c) transform oversampled data and according to Accuracy on (d) original, (e) naive oversampled, (f) transform oversampled data sets.

**Figure S5.** Stability for Multi-Label Classifier (ML) on the 606 sample set, finetuned in 20 epochs according to Matthews Correlation Coefficient on (a) original, (b) naive oversampled, (c) transform oversampled data and according to Accuracy on (d) original, (e) naive oversampled, (f) transform oversampled data sets.

**Figure S6.** Confusion Matrix of ML model, trained on 606 samples, of naive oversampled dataset, showing observed vs predicted taxonomic group. Prediction on test data for taxonomic ranks (a) phylum, (b) class, and (c) order.

**Figure S7.** Confusion Matrix of HC model trained on 606 samples of naive oversampled dataset, showing observed vs predicted taxonomic group. Prediction on test data for taxonomic ranks (a) phylum at epoch 20, (b) class at epoch 20, and (c) order at epoch 6.

**Figure S8.** LIME explanation for *Penicillium araracuarense* predicted with ML at epoch 4, with neighborhood size 1000 and 100 superpixels. Segmentation is performed by quickshift algorithm with kernelsize 6, max distance 50 and ratio 0.5. (a) Image of the colony without explanations, listed are label and prediction with score denoting the percent of correct predictions. LIME explanations are only provided for correct predictions. Black highlighted areas are LIME explanations at rank (b) phylum, (c) class, (d) order, (e) family, (f) genus.

**Figure S9.** (a) Number of unique taxa/classes according to taxonomic rank for **896 samples**. (b-g) Frequency distribution within each taxonomic rank. C is the number of categories, MC+ is the majority class count, MC- minority class count. A category is regarded as majority if the number of samples # is higher than the average number of samples per category. LriD is the likelihood ratio imbalance degree. For phylum, class, and order levels the majority classes are labeled for visualization.

**Figure S10.** Performance for Separate Local per-level classifiers (SL) finetuned in 20 epochs on **896 samples** according to Matthews Correlation Coefficient on (a) original, (b) naive oversampled, (c) transform oversampled data and according to Accuracy on (d) original, (e) naive oversampled, (f) transform oversampled data sets.

**Figure S11.** Performance of Multi-Label classifier (ML) finetuned in 20 epochs on **896 samples** according to Matthews Correlation Coefficient on (a) original, (b) naive oversampled, (c) transform oversampled data and according to Accuracy on (d) original, (e) naive oversampled, (f) transform oversampled data sets.

**Figure S12.** Hierarchically-Chained Local per-level classifier (HC) finetuned in 20 epochs on **896 samples** according to Matthews Correlation Coefficient on (a) original, (b) naive oversampled, (c) transform oversampled data and according to Accuracy on (d) original, (e) naive oversampled, (f) transform oversampled data sets.
