# Supplementary figures and images for: Deep learning image analysis for filamentous fungi taxonomic classification: Dealing with small datasets with class imbalance and hierarchical grouping
Source: Biol Methods Protoc. 2024 Aug 27;9(1):bpae063. doi: 10.1093/biomethods/bpae063 (PMC11387011; doi:10.1093/biomethods/bpae063)

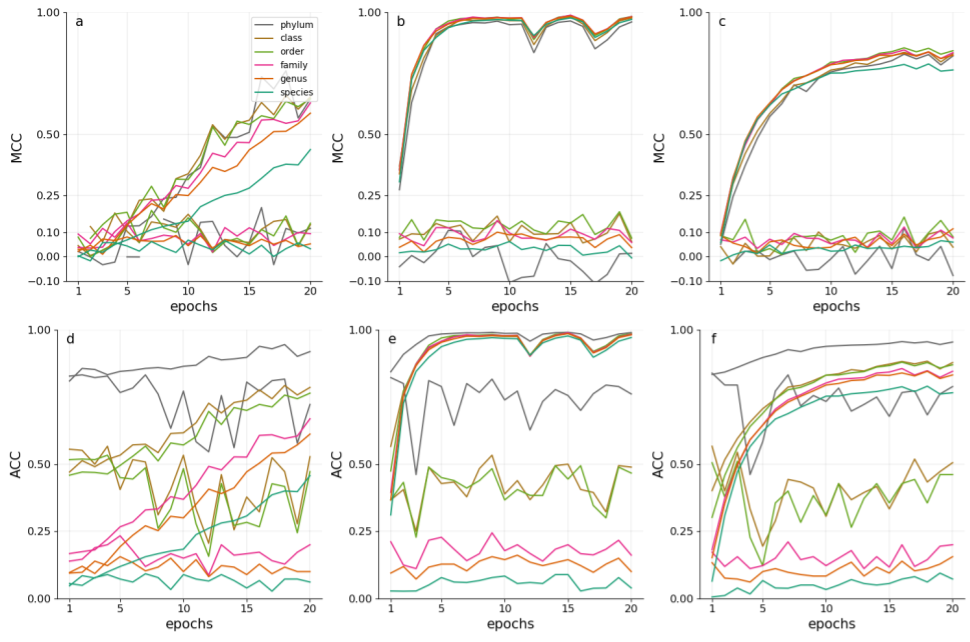

Supplement: bpae063_Supplementary_Data [file bpae063_supplementary_data.zip › Figure_S2.tif]

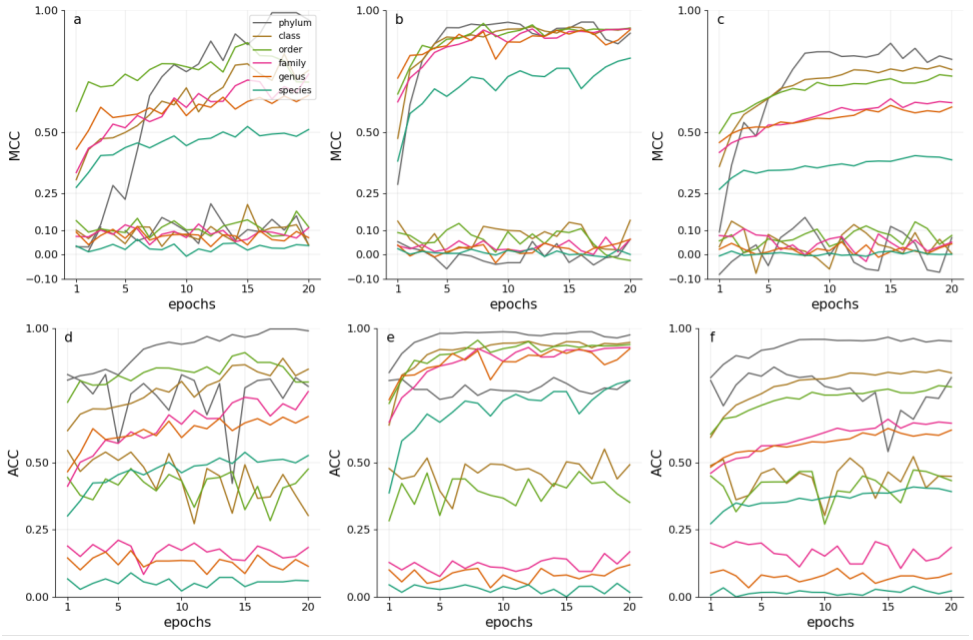

Supplement: bpae063_Supplementary_Data [file bpae063_supplementary_data.zip › Figure_S3.tif]

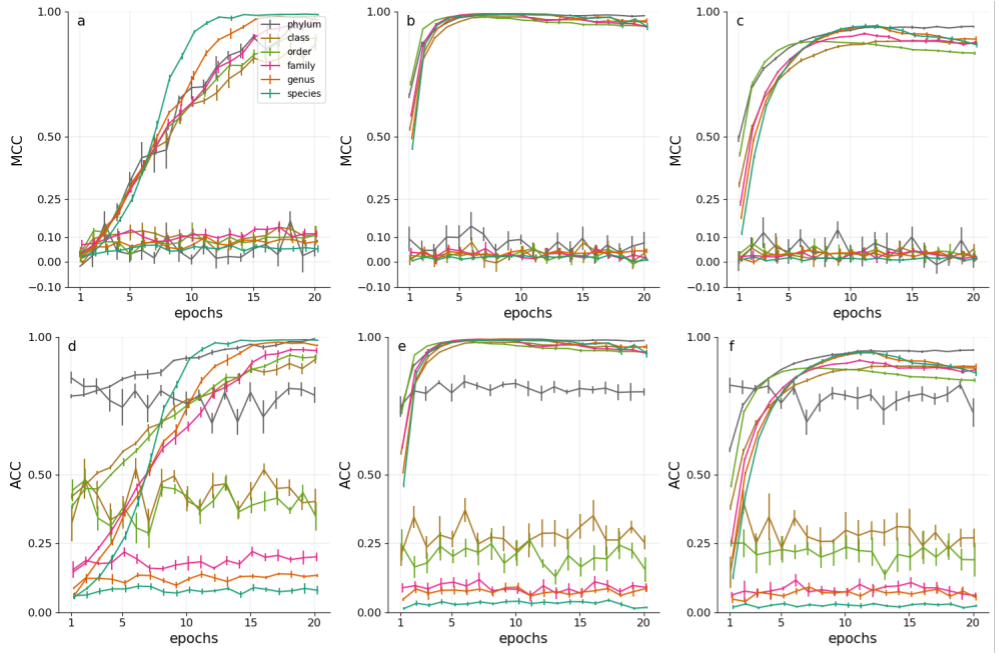

Supplement: bpae063_Supplementary_Data [file bpae063_supplementary_data.zip › Figure_S4.tif]

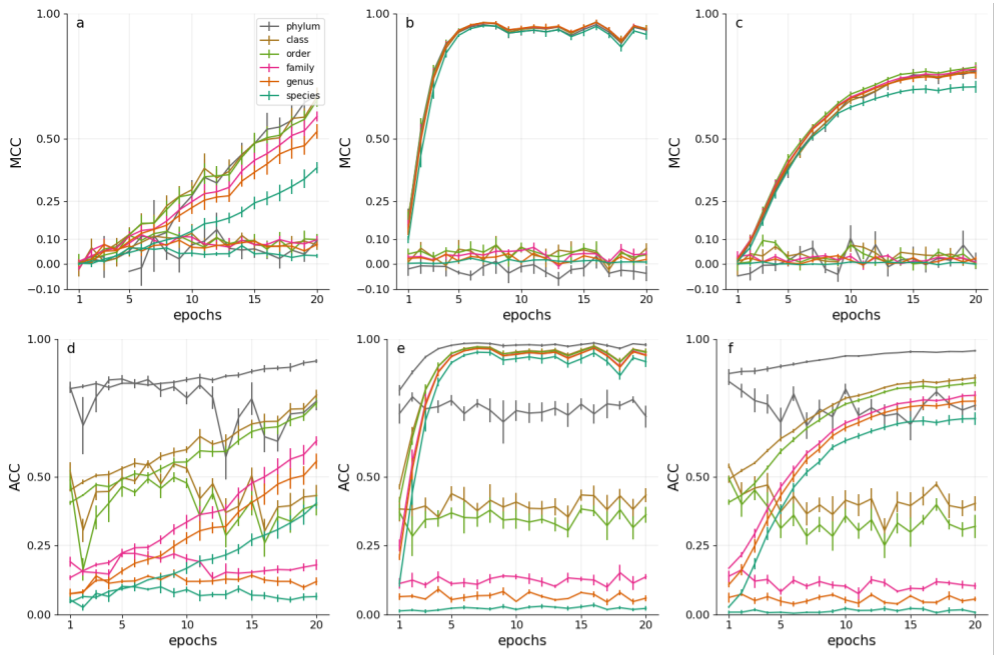

Supplement: bpae063_Supplementary_Data [file bpae063_supplementary_data.zip › Figure_S5.tif]

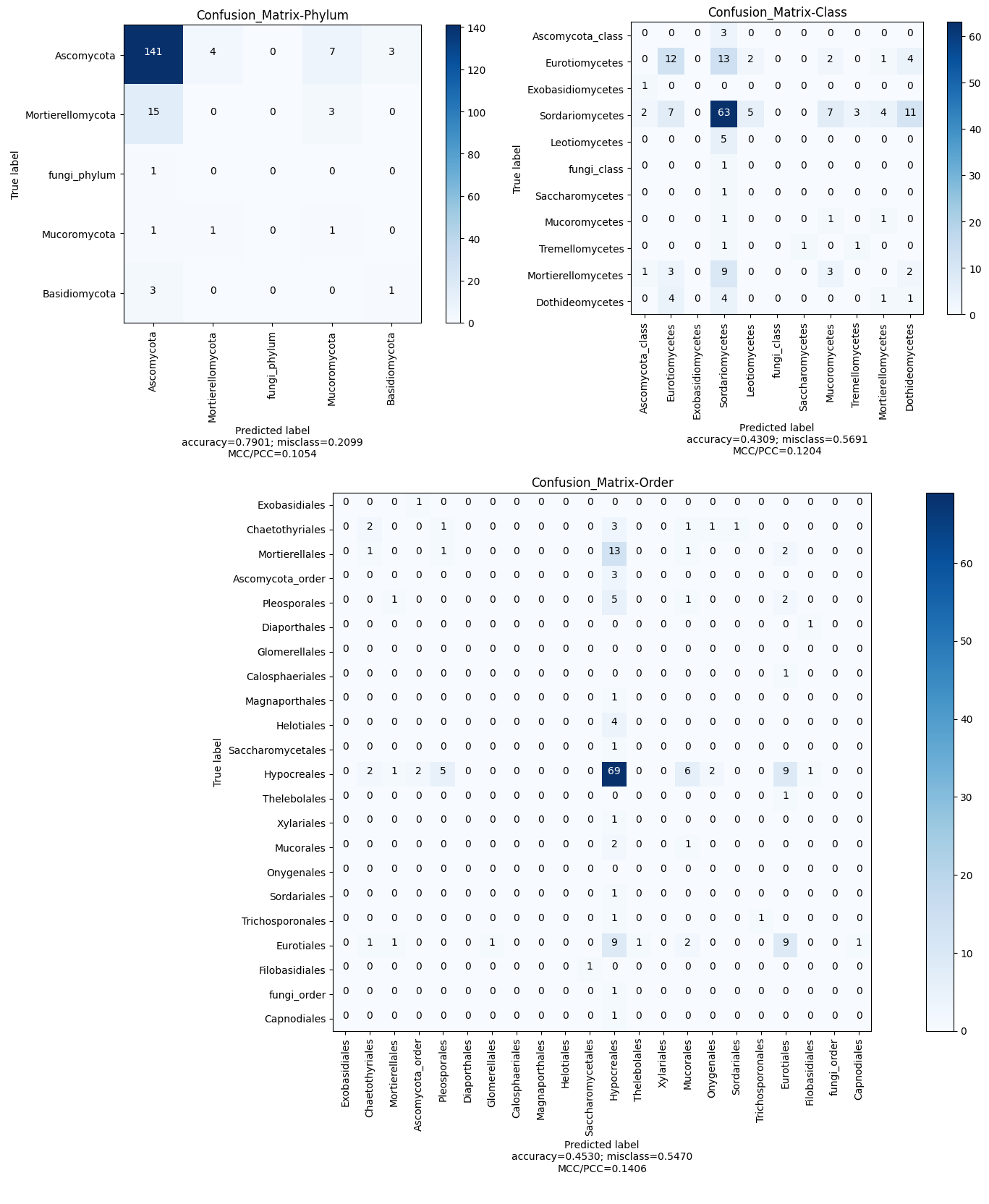

Supplement: bpae063_Supplementary_Data [file bpae063_supplementary_data.zip › Figure_S6.tif]

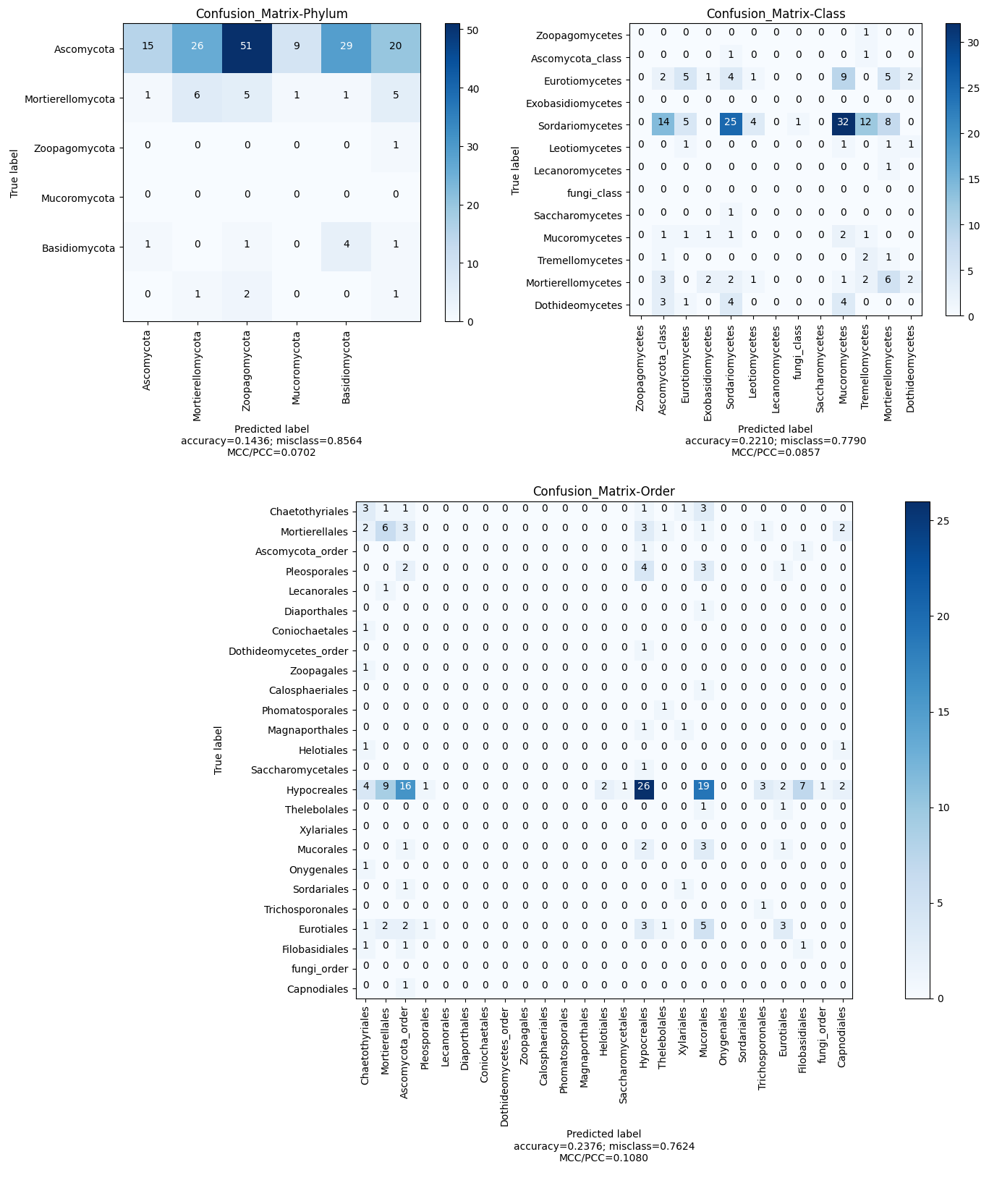

Supplement: bpae063_Supplementary_Data [file bpae063_supplementary_data.zip › Figure_S7.tif]

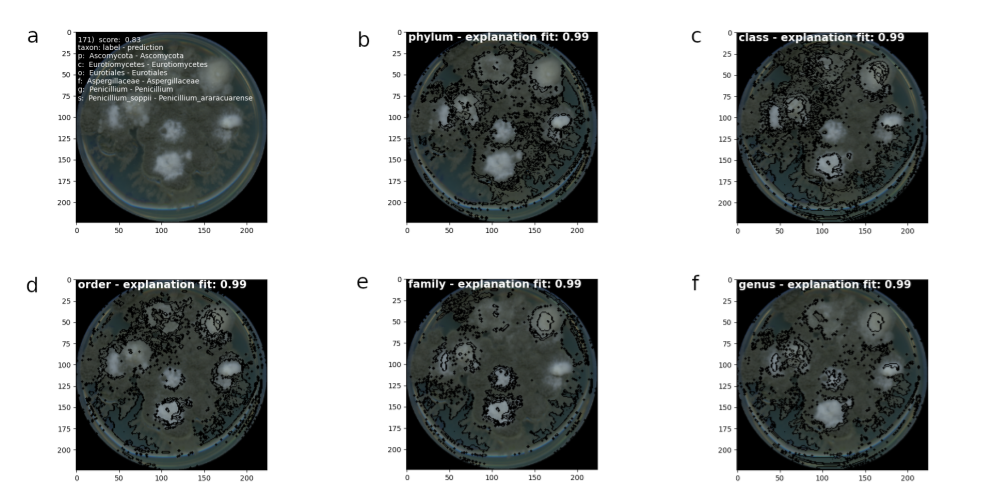

Supplement: bpae063_Supplementary_Data [file bpae063_supplementary_data.zip › Figure_S8.tif]

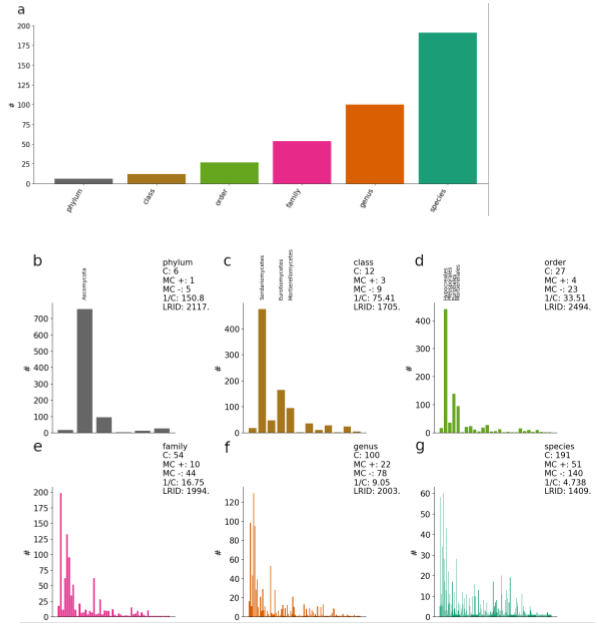

Supplement: bpae063_Supplementary_Data [file bpae063_supplementary_data.zip › Figure_S9.tif]

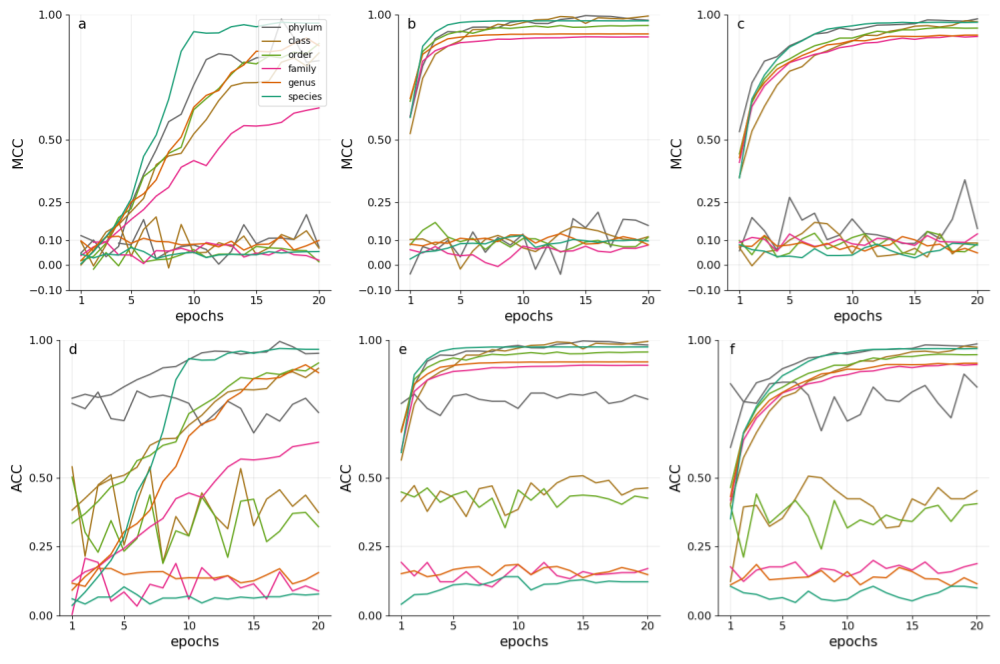

Supplement: bpae063_Supplementary_Data [file bpae063_supplementary_data.zip › Figure_S10.tif]

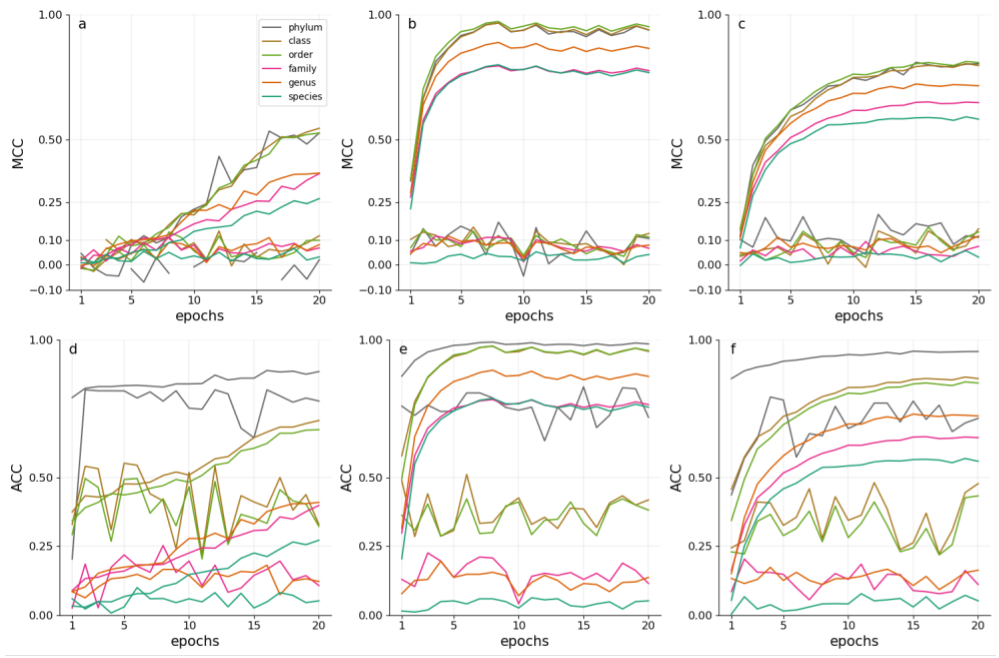

Supplement: bpae063_Supplementary_Data [file bpae063_supplementary_data.zip › Figure_S11.tif]

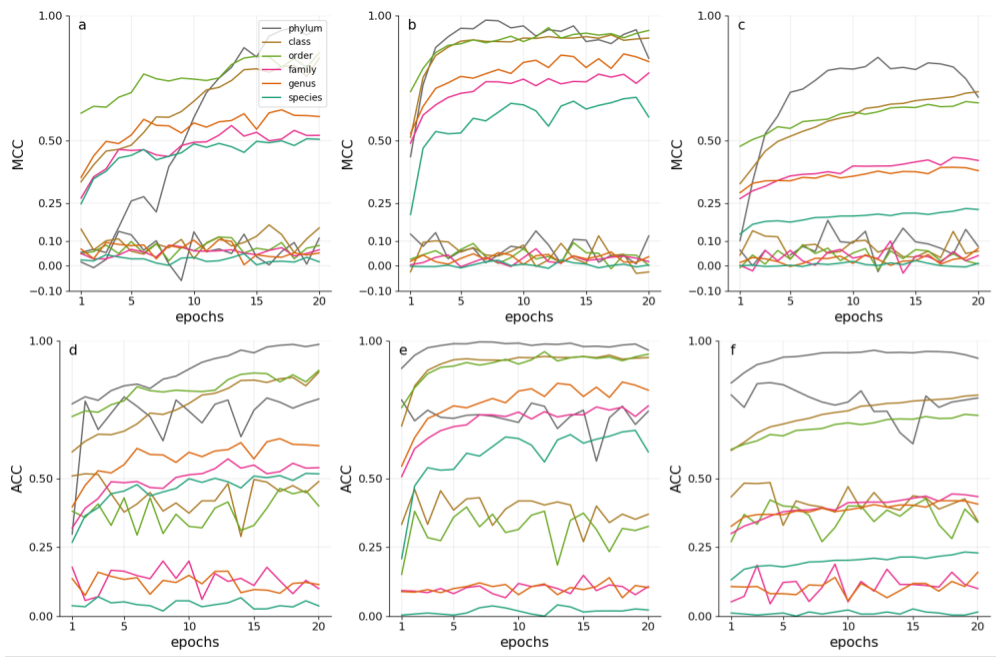

Supplement: bpae063_Supplementary_Data [file bpae063_supplementary_data.zip › Figure_S12.tif]

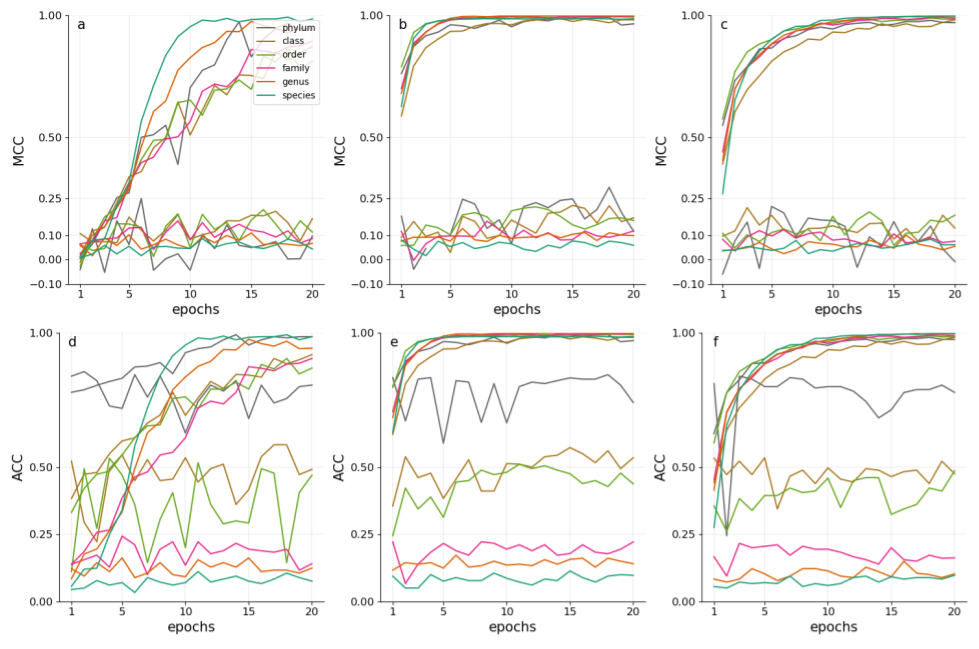

Supplement: bpae063_Supplementary_Data [file bpae063_supplementary_data.zip › Figure_S1.tif]
